# Does waterfall aerosol influence mucosal immunity and chronic stress? A randomized controlled clinical trial

**DOI:** 10.1186/s40101-016-0117-3

**Published:** 2017-01-13

**Authors:** Carina Grafetstätter, Martin Gaisberger, Johanna Prossegger, Markus Ritter, Predrag Kolarž, Christina Pichler, Josef Thalhamer, Arnulf Hartl

**Affiliations:** 1 Institute of Ecomedicine, Paracelsus Medical University, Strubergasse 22, 5020 Salzburg, Austria; 2 Institute of Physiology and Pathophysiology, Paracelsus Medical University, Strubergasse 22, 5020 Salzburg, Austria; 3 Gastein Research Institute, Paracelsus Medical University, Strubergasse 22, 5020 Salzburg, Austria; 4 Department for Radon Therapy Research, Ludwig Boltzmann Cluster for Arthritis and Rehabilitation, Strubergasse 22, 5020 Salzburg, Austria; 5 Institute of Physics, University of Belgrade, Pregrevica 118, 11080 Belgrade, Serbia; 6 Department of Molecular Biology, University of Salzburg, Hellbrunner Str. 34, 5020 Salzburg, Austria

**Keywords:** Ionized water aerosol, Waterfall, High-altitude climate therapy, Chronic stress, Green exercise, Burnout prevention, Mucosal immune response, Mountain hiking, Alpine space

## Abstract

**Background:**

The specific microclimate of alpine waterfalls with high levels of ionized water aerosols has been suggested to trigger beneficial immunological and psychological effects. In the present three-armed randomized controlled clinical study, we focused on effects on (i) immunological reagibility, on (ii) physiological stress responses, and on (iii) stress-related psychological parameters.

**Methods:**

People with moderate to high stress levels (*n* = 65) spent an active sojourn with daily hiking tours in the National Park Hohe Tauern (Großkirchheim, Austria). Half of the group was exposed to water aerosol of an alpine waterfall for 1 h/day (first arm, *n* = 33), whereas the other half spent the same time at a distant site (second arm, *n* = 32). A third arm (control, *n* = 26) had no intervention (except vaccination) and stayed at home, maintaining their usual lifestyle. The effect of the interventions on the immune system was tested by oral vaccination with an approved cholera vaccine and measuring specific salivary IgA antibody titers. Lung function was determined by peak expiratory flow measurement. Electric skin conductance, heart rate, and adaption of respiration rate were assessed as physiological stress parameters. Psychological stress-related parameters were analyzed by questionnaires and scales.

**Results:**

Compared to the control group, both intervention groups showed improvement of the lung function and of most physiological stress test parameters. Analysis of the mucosal immune response revealed a waterfall-specific beneficial effect with elevated IgA titers in the waterfall group. In line with these results, exposure to waterfall revealed an additional benefit concerning psychological parameters such as subjective stress perception (measured via visual analog scale), the Global Severity Index (GSI), and the Positive Symptom Total (PST).

**Conclusions:**

Our study provides new data, which strongly support an “added value” of exposure to waterfall microclimate when combined with a therapeutic sojourn at high altitude including regular physical activity.

## Introduction

Therapies at high altitude and green exercise are known to affect a variety of physiological and immunological parameters. These include neurovegetative, cardiovascular or thermoregulation mechanisms [1, 2], and also the reduction of inflammatory diseases, and the induction of balancing immunomodulatory effects [3–5].

High-altitude climate therapy is also characterized as a successful alternative medical treatment for respiratory and allergic diseases such as bronchial asthma, atopic dermatitis, psoriasis, or eczema [6–8].

In addition to high altitude, European alpine regions host numerous waterfalls, which produce inhalable, negatively charged nano-water particles known as “Lenard ions” or ballo-electric ions. Negative air ions nearby waterfalls (hereinafter we call them “ionosols”) are generated by aerosolization of water droplets at an obstacle, aqueous surface, or by aerodynamic break-up during free fall, undergoing charge redistribution forming “water bags” with negatively charged surfaces and fracturing into micro-bubbles due to water shearing. After breaking up, smaller fragments carry negative charge and remain in the air for some time carried by air stream. Their diameter is between 1.5 and10 nm, whereby 2-nm-sized negative ions were the most abundant. Lifetime of ionosols is long enough so that they can be inhaled. The remaining bigger fragments are positive and precipitate to the ground [9, 10].

“Negative air ions” are positively correlated with relative humidity and their concentration is higher in natural environments compared in urban environment [11–14]. Waterfalls not only produce high levels of negative air ions, they also create a specific microbiological atmosphere by spreading of microbes in the impact zone of a waterfall, which may influence immunological and physiological parameters. Negative air ions also interact with phytoncides released from trees which in turn can interact with microbes, altogether influencing the environmental atmosphere [14]. Phytoncides may also act directly on the innate immune system, as they have been shown to increase the level of natural killer cells [15].

Positive immunomodulating effects of the microclimate in close proximity to the impact zone of alpine waterfalls have already proven beneficial effects for the treatment of allergic asthma [16]. In this previous study, half of a group of asthmatic allergic children (*n* = 54) was exposed to water aerosol of an alpine waterfall for 1 h/day over 3 weeks, whereas the other half spent this hour at a water aerosol free control site. The exposure to water aerosol had a long-lasting beneficial effect on asthma symptoms and lung function, accompanied by a decreased inflammatory immune status as indicated by elevated levels of IL-10 and regulatory T cells.

Mechanisms underlying the immunomodulatory effect of high altitude include the normalization of eosinophil levels, balancing of the TH2/Treg cell ratio, increase of regulatory cytokines (e.g., IL-10), and even alteration of immunoglobulin class switching [3, 4, 16, 17]. Furthermore, alterations of adrenocorticotropic hormone (ACTH) and glucocorticoid secretion indicate that high altitude affects both neuronal as well as immune circuits [1]. Negative air ions are also associated with psychological well-being, e.g., due to an increased serotonin level [18]. Considering, it is conceivable that high-altitude climate may also affect stress and its pathologic immunological, physiological, and psychical consequences. Stress is known to induce endocrine changes via mediators such as glucocorticoid hormones and catecholamines and thus also to influence humoral and cellular immunity [19]. In a well-balanced but fragile molecular and cellular network, the immune, endocrine, and nervous system affect cognitive performance, well-being, and behavior, and, in general, also maintain health [20, 21]. For example, people with high occupational stress, like caregivers, show impaired specific and unspecific immune responses, both concerning humoral and cellular immunity [22, 23], and chronic stress is associated with a shift from a TH1 to a more TH2-type immune response cytokine pattern [24]. Similar T cell response types are also observed in the development of asthma, which is not only influenced by stress, anxiety, and depression but also affected by the microbiome [25]. Also, mucosal immune responses show a clear correlation with stress. As a consequence of chronic stress, the abundance of salivary IgA is decreased [26–29], and even more subtle psychological parameters (like disgruntlement) seem to influence salivary IgA levels [30–32]. As secretory IgA is present in all mucosal surfaces, it is of crucial importance for the first line of defense of the immune system [33, 34]. Therefore, monitoring specific antibodies in a workload situation, e.g., during a vaccine-induced immune response, represents a suitable model to estimate the immune status of an individual. Several studies demonstrate the strong impact of psychological stress on a developing humoral response, e.g., chronic burden (like nursing care of a sick member of the family) interferes with the humoral immune response to an influenza vaccination [33, 35–39].

Typical and useful physiological parameters indicative for stress and emotional strain are peripheral physiological signals such as electric skin conductance (SC), respiration rate (RSP), and heart rate (HR), all being measurable in response to an artificial stressor [40–42]. The conductance of the skin is dependent of the quantity of sweat, which is controlled by the sympathetic nervous system and positively correlates with stress. Also, respiration reflects the psychological and emotional state, e.g., in relaxed situations, respiration is slow and regular, whereas fast and irregular rhythms of breath are ascribed to stress emotions [43]. Similarly, the heart rate and its variability correlate with comforting impacts and defensive reactions. Stress and increased sympathetic activity normally lead to an increase of heart rate, whereas parasympathetic activity has the opposite effect [44, 45]; thus, effective stress adaption is known to increase general well-being [46].

Based on the data showing an immunomodulatory effect of the waterfall aerosols, we hypothesized that the waterfall microclimate may exert a positive effect on different stress parameters.

Therefore, the present randomized controlled clinical study investigated whether in combination with a high-altitude climate therapy, waterfall-generated ions would provide an effective and cost-efficient therapy to mitigate various stress-related symptoms and strengthening immunity.

As a trigger of many secondary diseases such as neuropsychiatric, cardiovascular, or metabolic disorders, stress induces enormous economic costs [47, 48]. Interestingly, nature itself may preserve a sustainable health resource to counteract this trigger.

## Methods

### Subjects

**Fig. 1:**
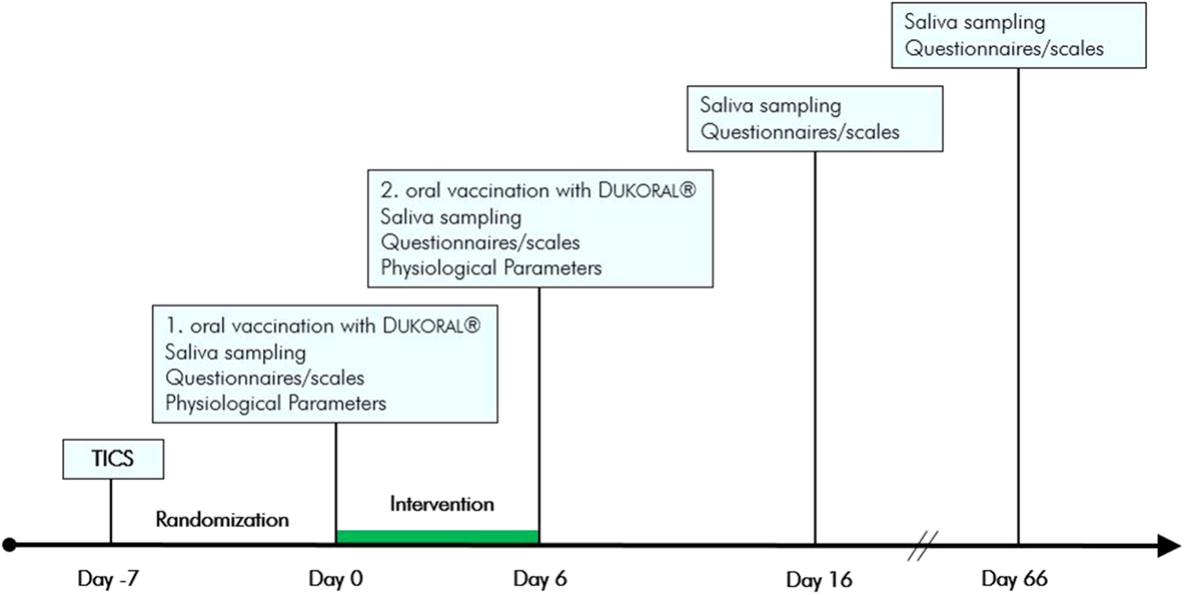
Study schedule. Schematic chronological process of the controlled clinical trial

Ninety-one persons working in care professions (19–61 years old; 44 men, 47 women) were enrolled in the study. People working in care professions are known as high-risk occupational groups concerning psychological and immunological consequences of stress [19, 24]. Requirement for study inclusion was moderate to high stress levels as defined by the *Trier Inventory for the Assessment of Chronic Stress (TICS)* questionnaire, assessed 7 days before beginning of the study (Fig. 1), and no previous cholera vaccination. Exclusion criteria were fever, chronic or severe diseases, including immune dysfunctions, and treatment with antibiotics or immunosuppressants. The study was approved by the ethical committee of Salzburg (E1066/29042009).

### Study design/intervention

The study was set up as a randomized controlled clinical trial with three arms.

Except for the non-intervention (control) group (CO, *n* = 26), all participants spent 1 week at the village of Großkirchheim (Carinthia, Austria) located 1024 m above sea level, hosted in hotels and receiving the same meals. For 1 h each day, the groups were separated for intervention into a waterfall- (WF+, *n* = 33) and a “non-waterfall” group (WF−, *n* = 32); group separation was kept identical throughout the study. Individuals of the control group stayed at home (without any intervention, except vaccination), following their usual daily life.

The probands of the WF+ group were exposed for 1 h to the Gartl Waterfall at 1000 m a.s.l., whereas the WF− group spent this hour at a site about 1000 m away from the waterfall, but at the same altitude. The average concentration of ionosols at this “control” site was 840 ions/cm^3^ (mean air humidity 72.3% ±23.1, mean temperature 15.3 °C ±2.5), compared to the average 42660 ions/cm^3^ with maxima up to 57510 ions/cm^3^ at the waterfall exposed site (mean air humidity 84.6% ±9.9, mean temperature 16.9 °C ±4.1) [9]. In order to exclude effects based on different trainings, this location was selected at the same altitude as the waterfall, with identical cumulative elevation gain and distance from the starting point.

Except for this 1-h intervention, participants of the WF+ and WF− group had the same daily routine, and both groups went through a daily hiking program at an altitude between 1400 and 2900 m, with structured hikes of at least 600 meters of cumulative elevation gain.

All participants received an oral cholera vaccine on day 0 and day 6 of the study (Fig. 1).

The study took place from July to August 2009; follow-up examinations were performed after 2 months (Fig. 1). Physiological and psychological data from day 0 and day 6 were evaluated on site in a medical field laboratory in Großkirchheim, Carinthia. Follow-up examinations on day 16 and day 66 were held in the Paracelsus Medical University Salzburg. Randomization was performed with “Random allocation software” program [49].

### Environmental parameters of the Gartl Waterfall

The waterfall is located in the heart of the National Park Hohe Tauern in Carinthia (Austria), situated in the community Großkirchheim in the upper Möll Valley. It is easily accessible via a gently rising 1.5-km walk from the village center and well established for waterfall exposition. Its approximate drop height is 50 m in two cascades and the average water flow is 0.6 m^3^/s (max 0.9 m^3^/s). Despite the moderate drop height, the waterfall features very high concentrations of ionosols (mean 42660 ions/cm^3^, max 57510 ions/cm^3^) floating the whole valley beneath the impact zone. Air humidity near the impact zone is 60–90%, consisting of respirable small water drops, whereas ozone levels are virtually zero.

### Vaccination

Mucosal immunity was tested by oral vaccination with DUKORAL® from SBL Vaccines, which was given on days 0 and 6 (Fig. 1). Dosage and mode of application were according to the package information leaflet [50, 51].

### Collecting and analysis of saliva

Saliva (2–3 ml) was collected from all subjects in sterile 15-ml plastic tubes on days 1, 6, 16, and 66 in the morning (Fig. 1), immediately after waking up (thus containing the matutinal IgA concentration peak) [52]. The tubes were stored at –80 °C until preparation and analysis. To determine DUKORAL*®* vaccine specific salivary IgA concentrations, 96-well high-bind, flat-bottomed immunoplates (FluoroNunc, Roskilde, Denmark) were coated overnight at 4 °C with DUKORAL® vaccine antigens (1 mg recombinant cholera toxin B subunit (rCTB)) diluted 1:100 with PBS (1×), corresponding to 0.01 mg per well. After blocking for 1 h at room temperature with 200 μl/well PTB (PBS/0.1% Tween 20/0.5% bovine serum albumin (BSA)), the plates were washed with PBS/0.1% Tween 20, and saliva was added at a dilution of 1:100 in 100 μl/well PTB for specific IgA and incubated for 1 h at RT. The plates were washed with PBS/0.1% Tween 20 and incubated with 100 μl/well horseradish peroxidase (HRPO)-conjugated goat anti-human IgA (BioRad, Austria), diluted 1:1000 in PTB for 1 h at RT. Again, the plates were washed (with PBS) and the reaction was detected by adding luminol (Boehringer Mannheim, Germany) (5 ml solution A (100 mM glycine pH 10 (NAOH)/0.4 mM luminol/8 mM iodophenol), 5 mL solution B (0.12% H_2_O_2_ in dH_2_O), and 10 ml dH_2_O). Chemiluminescence was measured by a Promega ELISA-plate luminometer (Promega, Mannheim, Germany). Luminescence was calculated as photon counts per second.

### Lung function and physiological stress test

The respiratory parameter analyzed was peak expiratory flow (PEF) by means of a forced ex-in maneuver. All tests were performed with EasyOneTM Plus Diagnostic from ndd Medical Technologies by aid of two trained scientists.

A computer-guided physiological stress test was performed to measure heart rate (three-point ECG), respiration rate (respiration sensor), and skin conductance (finger clip) during a 3-min baseline phase, a 1-min stressor phase (optical and acoustical stressors on the computer screen), and a 4-min post-stress recovery phase using a NeXus 10 multimodal device from Mind Media (Mind Media BV, Netherlands). The arithmetic mean of the respective physiological signal during the stressor phase was set to 100%. The arithmetic mean of the post-stress recovery phase was compared to the 100% line and used to measure the stress adaption of the peripheral signals heart rate, respiration rate, and skin conductance.

### Questionnaires and scales

#### Trier Inventory for the Assessment of Chronic Stress

This questionnaire was used to differentiate between various facets of chronic stress, measured by the retrospective rate of stress events in six areas (worries, work overload, work discontent, lack of social recognition, social stress, and intrusive memories) on a five-point rating scale [53].

#### Visual analog scale

VAS was performed as a measurement tool for subjective stress perception. On basis of a linear scale, participants can state their current stress level by indicating a position along a continuous line between the two end-points “no stress at all” and “unbearable stress”. The analog aspect of linear scales creates preferably metrical characteristics instead of discrete scales [54].

#### Symptom Check List–SCL 90

The SCL 90 questionnaire enables to analyze treatment or progress of a variety of symptom dimensions, such as somatization, obsessive-compulsive, interpersonal sensitivity, depression, anxiety, hostility, phobic anxiety, aggression, paranoid ideation, and psychoticism. The test provides an overview and displays intensity of symptoms at the moment and in progress, including three global indices, i.e., Global Severity Index (GSI) (describing overall psychological distress), Positive Symptom Distress Index (PSDI) (describing intensity of symptoms) and Positive Symptom Total (PST) (describing the number of self-reported symptoms) [55].

#### Maslach Burnout Inventory (MBI-D^©^)

The original Maslach Burnout Inventory was developed to measure intensity and incidence of perceived burnout in care professions. The authorized German version MBI-D, closely aligned with the original, surveys three components of burnout syndrome based on several specific items: emotional exhaustion, depersonalization, and personal accomplishment [56].

#### List of complaints (KSb–S BL)

This questionnaire is an independent part of the test-battery KSb–S (the german abbreviation KSb–S stands for “clinical self-assessment-scales”, BL for “list of complaints”, PSYCHIS Munic), which captures the degree of subjective impairment due to physical, e.g., joint pains and general complaints (e.g., faintness). Combination of quantity and levels of manifestations (heavy–moderate–little–not at all) result in a total interference of complaints, reflecting a constitutional change over time [57].

#### Mental state scale (KSb–S Bf–S)

Like the BL, the Bf–S is also an autonomous part of the test-battery KSb–S which is arranged to reflect the change of the present, psychical, and subjective mental state in an aggregate value, figuring the whole range of normal and pathologic changes of well-being [58].

#### Recovery-Stress Questionnaire–EBF

This questionnaire figures an individual’s current recovery-stress state using retrospective (last 3 days) information of stressful situations, reactions, and recovery activities in a self-assessment test. Answers are queried in a seven-point scale, ranging from never to always [59].

#### Statistical analysis

All analyses were performed using the IBM SPSS statistics version 22 (IBM, NY, USA, http://www.spss.com/). Different linear mixed models, three-armed (LMM1, WF+, and WF− group compared to CO group) and two-armed (LMM2, WF+ group compared to Wf− group) were used for statistical analysis of all variables with more than two time points and high individual variations. Unlike a linear regression analysis, a linear mixed model is able to detect changes despite high individual variations in the IgA levels and questionnaires. Time, treatment, and interaction of treatment and time were set as fixed factors. To take individual differences into account, the patient IDs were included in the models as random effect. A third linear mixed model (LMM3) was used to detect changes over time for the main outcome variable of secretory IgA (sIgA) levels, excluding the interaction of time and treatment, with only time and treatment as fixed factors of each. Linear regressions were performed to detect intervention effects in all physiologic parameters and the visual analog scale, lacking of high-individual variations and inset at two time points. To identify significant differences of the baselines between the three study groups, comparison of means was done with ANOVA including Bonferroni correction for parametric data; Wilcoxon signed-rank test or Mann–Whitney *U* test was applied to non-parametric data.

Figures are shown as means ± standard deviation (SD). Statistical significance was expressed as *p* ≤ 0.05 (*) or as *p* ≤ 0.01 (**).

Furthermore, all study data were subjected to Kolmogorov–Smirnov (with Lilliefors correction of significance) and Shapiro–Wilk normality tests—70% of the data generated passed the tests.

## Results

### Patient characteristics

The randomized, controlled clinical study comprised 91 participants working in care professions consisting of 47 females (mean BMI 22.50 ± 3.51 SD) and 44 males (mean BMI 24.33 ± 3.16 SD), ranging from 19–61 (30.93 mean ± 10.39 SD) years of age. No significant differences were found between baseline values of both intervention groups (WF+ and WF– group). The control group (CO) had a bias in age and was slightly younger compared to both other groups.

### Response to mucosal vaccination

The vaccination protocol led to a detectable antigen-specific mucosal immune response in approximately one half of the immunized subjects. Responders and non-responders were defined by measuring vaccine-specific salivary sIgA titers. A value of three standard deviations above the IgA titer of the serum taken prior to immunization (pre-serum) served as baseline. Participants with values above baseline were determined as responders (∑*n* = 48 (*n* (WF+) = 16; *n* (WF–) = 18; *n* (CO) = 14)). Data of non-responders (∑*n* = 43, equates ~47%) got excluded from statistical analysis of specific IgA levels.

**Fig. 2:**
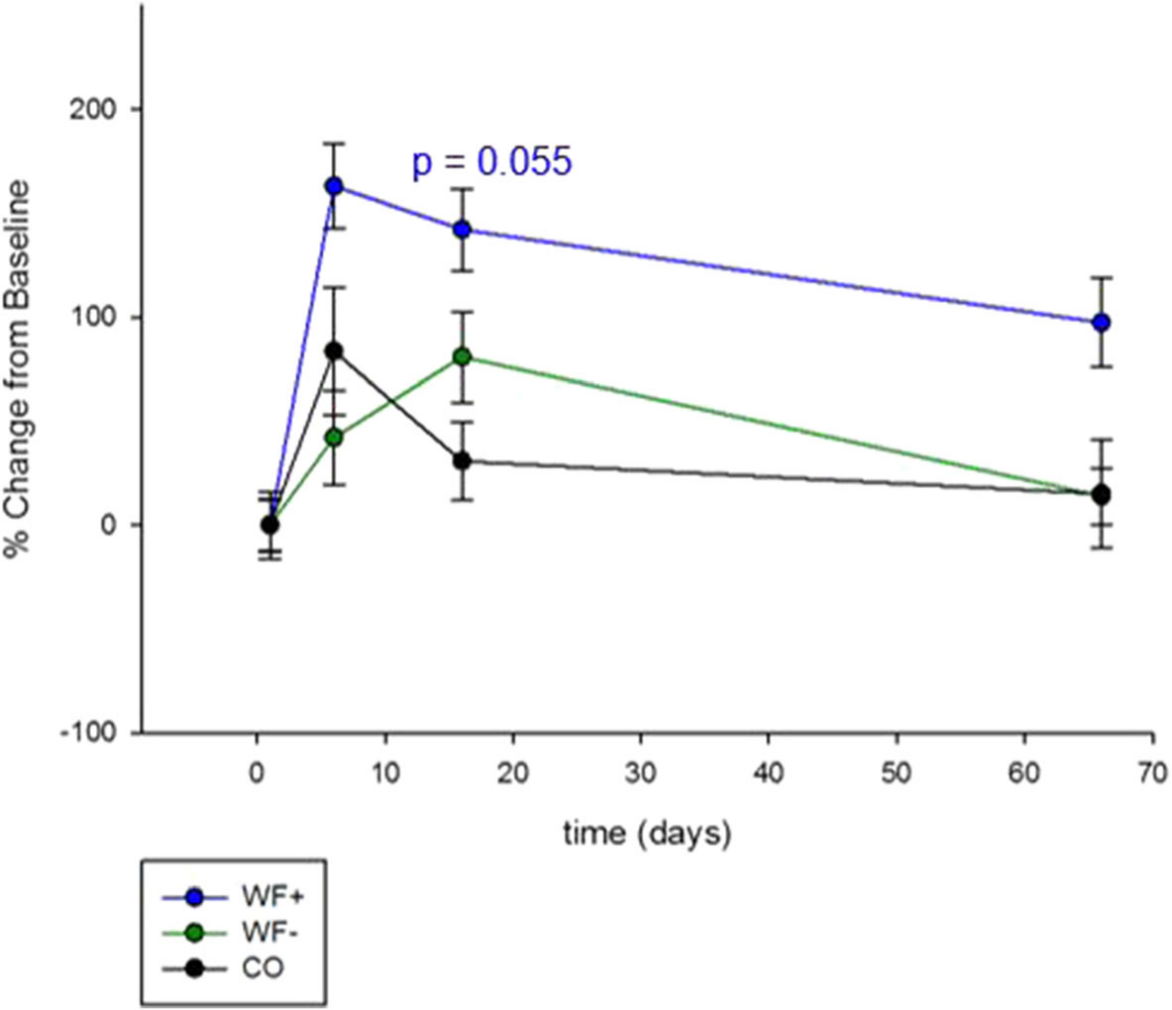
IgA levels of all groups. Antigen-specific salivary IgA levels of responders. Linear mixed model (LMM1) of both intervention groups compared to the control group. Significance are indicated by *p* values. Data are shown in percentage change (±SD) from baseline (pre-serum)

A comparison of IgA titers of both intervention groups (WF+ and WF–) with the non-intervention control group with a linear mixed model (LMM1) shows no significant difference but a statistical trend (*p* = 0.055) on day 16 of the WF+ group, compared to that of the CO group (Fig. 2). The strongest percentage increase at all time points can be found in the WF+ group (Table 1).

**Table 1:** Antigen-specific salivary IgA-levels

		Day 6	Day 16	Day 66
WF+	Photon counts	498 583.8	458 859	374 034.3
	% change	163.1	142.1	97.4
WF−	Photon counts	365 445.3	465 156.6	293 012.7
	% change	42.2	80.9	14
CO	Photon counts	615 312.5	438 061.5	385 653.7
	% change	83.6	30.7	15.1

Data of the intervention groups (WF+ and WF-) and the control group are given in photon counts. % change indicates the increase of IgA titers after immunization compared to the baseline (pre-serum)

**Fig. 3:**
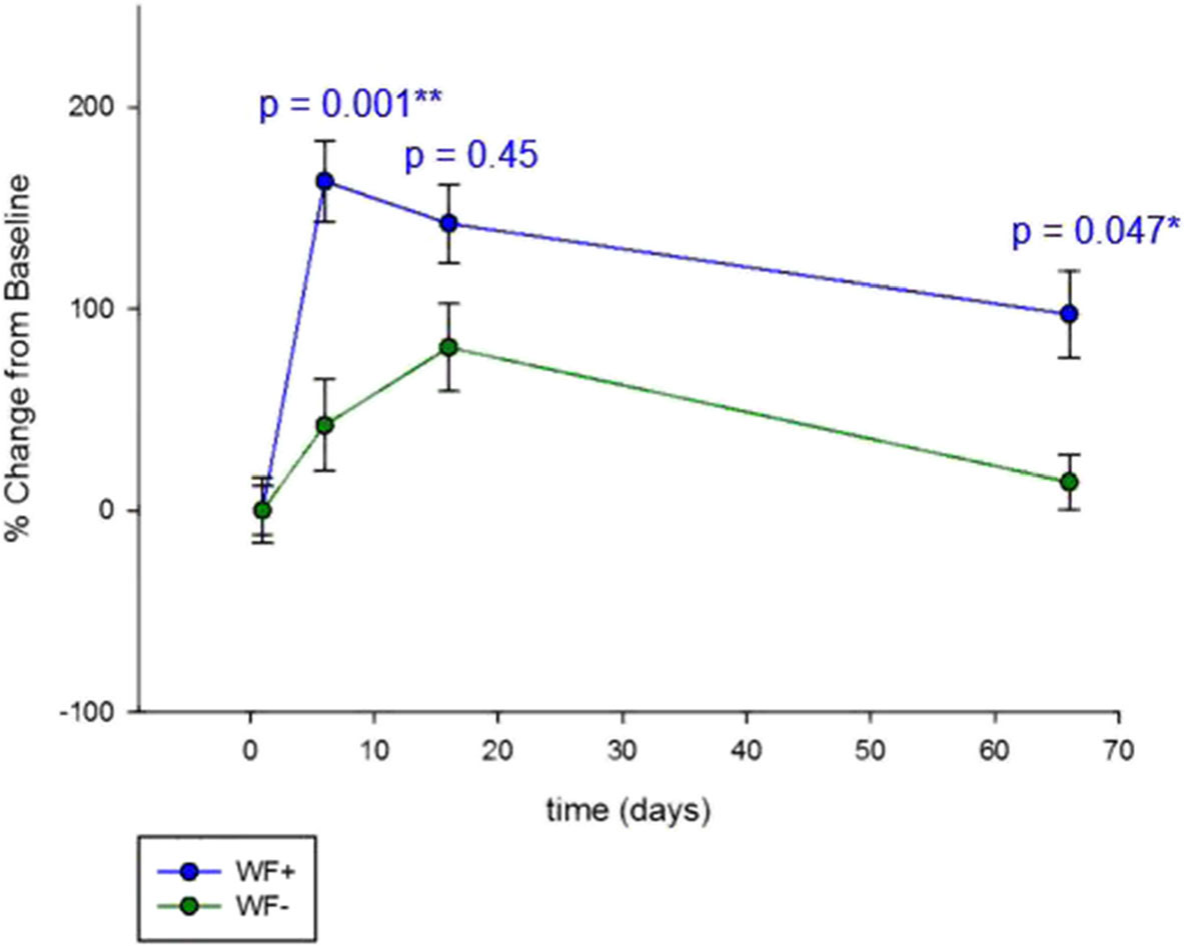
IgA levels of the two intervention groups. Antigen-specific salivary IgA levels of responders. Linear mixed model (LMM2) of the waterfall group (WF+) compared to the non-waterfall group (WF−). Significances are indicated by *p* values. Data are shown in percentage change (±SD) from baseline (pre-serum)

The specific impact of the waterfall ionosols was evaluated with a linear mixed model (LMM2) of the two intervention groups WF+ and WF–. The result indicates a significant increase of salivary IgA titers in the WF+ group on day 6 and day 66 (Fig. 3).

A linear mixed model excluding interaction of time and treatment (LMM3) showed significant changes over time in both intervention groups at all time points (day 6 *p* = 0.000, day 16 *p* = 0.000, day 66 *p* = 0.001).

### Subjective stress perception

**Fig. 4:**
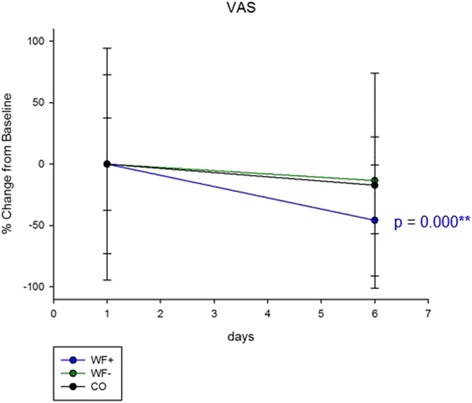
Subjective stress perception. Visual analog scale of subjective stress perception. Stress levels in the WF+ group were significantly decreased (compared to control) and shown a trend to a specific waterfall effect (WF+ to CO *p* = 0.000, *R* ^2^ = 0.615; WF+ to WF−: *p* = 0.054, *R* ^2^ = 0.179; WF– to CO *p* = 0.066, *R* ^2^ = 0.496). Data shown in means (±SD). Statistical analysis calculated with linear regression

The visual analog scale assessing the subjective stress perception was applied on day 1 and day 6. A significantly lower stress level could be measured in the WF+ group after the intervention week on day 6, compared in the control group (lin. regression, *p* = 0.000, *R* ^2^ = 0.615), and a clear trend to a waterfall-specific beneficial influence when compared to the WF– group (lin. regression, *p* = 0.054, *R* ^2^ = 0.179) (Fig. 4).

### Psychological stress parameters and burnout symptoms

**Fig. 5:**
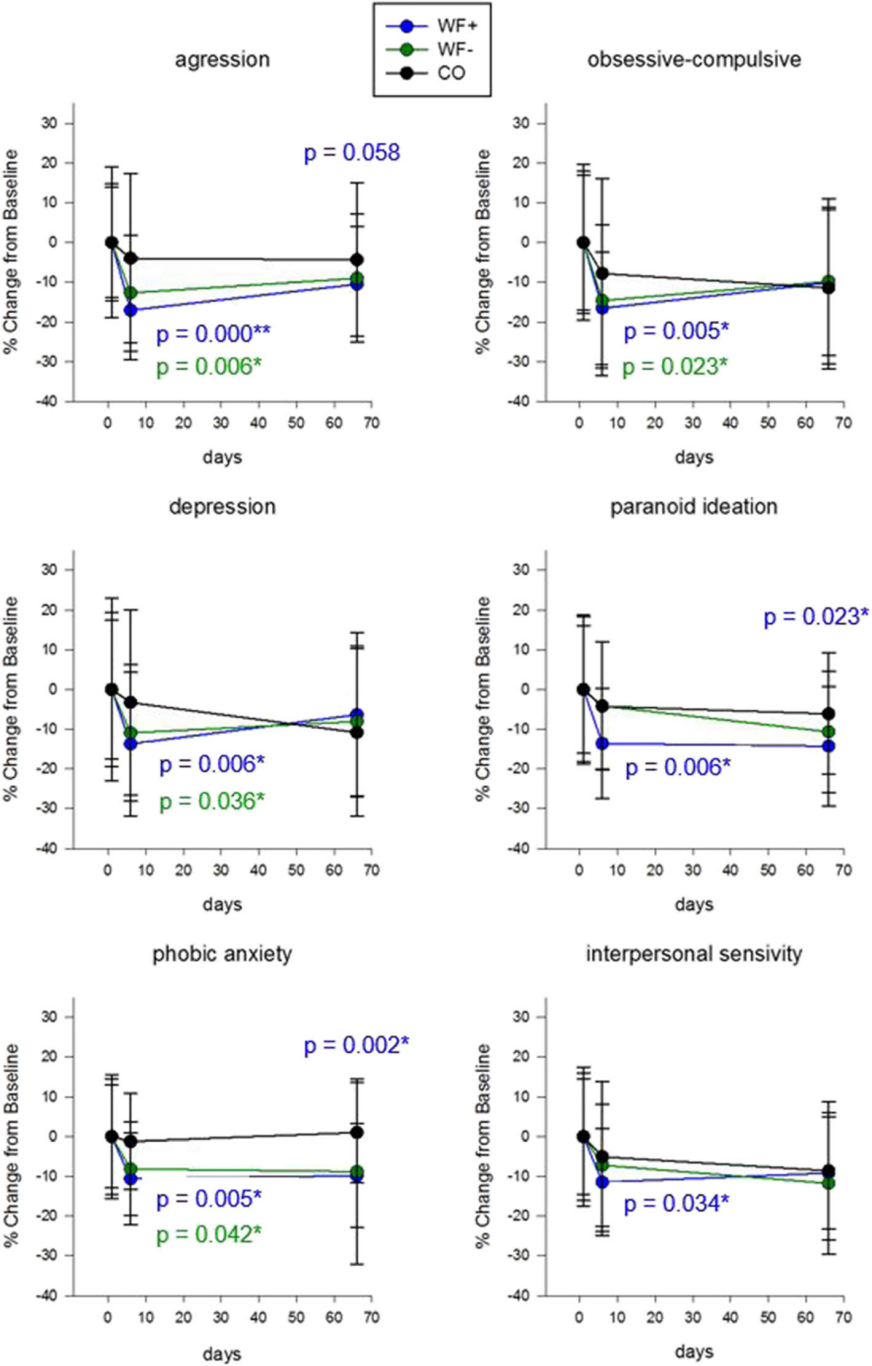
SCL-90 questionnaire. Results of the SCL-90 questionnaire shown in percentage change from baseline. The data indicate beneficial effects of both interventions, with stronger significances for the waterfall group. Linear mixed models of the three study groups over time. Data shown in means (±SD)

Linear mixed model (LMM1) analysis of the SCL-90 questionnaire revealed improvement in six of the ten symptom dimensions in both intervention groups after 6 days (compared in the control group): aggression, day 6: WF+ *p* = 0.000; WF– *p* = 0.006, day 66: WF+ *p* = 0.058; obsessive-compulsive, day 6: WF+ *p* = 0.005; WF– *p* = 0.023; depression, day 6: WF+ *p* = 0.006; WF− *p* = 0.036; paranoid ideation, day 6: WF+ *p* = 0.006, day 66: WF+ *p* = 0.023; phobic anxiety, day 6: WF+ *p* = 0.005; WF− *p* = 0.042, day 66: WF+ *p* = 0.002; interpersonal sensitivity, day 6: WF+ *p* = 0.034 (Fig. 5). No differences could be detected with respect to the parameters somatization, anxiety, hostility, and psychoticism (data not shown) [55]. Significant long-lasting effects (day 66) could be measured in the waterfall group for two parameters (phobic anxiety and paranoid ideation). Again, the data indicate a clear trend of a beneficial effect of the waterfall environment.

**Fig. 6:**
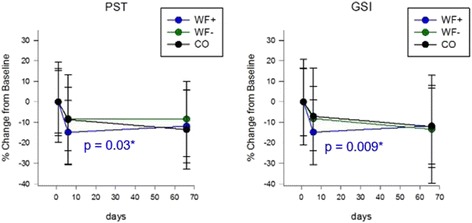
Global Severity Index and Positive Symptom Total Score of the SCL-90. Global indices of the SCL-90 questionnaire. The data show significant improvements of the Global Severity Index and the Positive Symptom Total score (PST *p* = 0.03, GSI *p* = 0.009) in the WF+ group on day 6. Data shown in percentage change from baseline (±SD)

Furthermore, on day 6, both the Global Severity Index assessing overall psychological distress, as well as the Positive Symptom Total, which displays the number of self-reported symptoms, were significantly decreased in the waterfall group only (*p* = 0.009 for GSI and *p* = 0.03 for PST), again indicating a waterfall-specific effect (Fig. 6). The Positive Symptom Distress Index, which describes the intensity of symptoms, elicited no differences over time between the three study groups (data not shown).

Measuring incidence and severity of burnout by means of the *Maslach Burnout Inventory* (MBI-D) elicited a significant improvement in the WF+ group in the category *depersonalization*, compared in the control group on day 66 (day 6 *p* = 0.064, day 66 *p* = 0.002). No differences could be measured concerning the other two components, i.e., *emotional exhaustion* and *personal accomplishment* over time in all groups. With respect to the list of complaints (BL), reflecting constitutional changes during the trial, the WF+ group showed a significant positive long-term effect on day 66 (*p* = 0.011). Both intervention groups show a decrease of complaints on day 16 (WF+ *p* = 0.086, WF− *p* = 0.057), and the mental state scale (Bf–S) clearly indicates enhanced well-being short-term effects on day 6 (WF+ *p* = 0.051, WF− *p* = 0.06). The recovery and stress questionnaire (EBF) revealed less stress (day 6, *p* = 0.068) and improved recovery only in the waterfall group (day 6 *p* = 0.07, day 66 *p* = 0.036). All statistical analyses were done with LMM1 (data not shown).

### Lung function and stress-associated peripheral physiological parameters

**Fig. 7:**
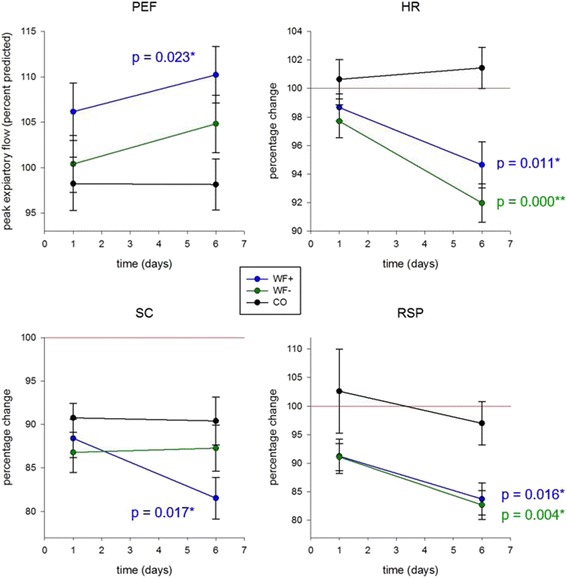
Lung function and physiological stress test. Linear regression analysis of lung function and peripheral signals of the physiological stress test. Spirometry was performed measuring peak expiratory flow (PEF), as peripheral signals of physiological stress served heart rate (HR), skin conductance (SC), and respiration rate (RSP). Means (±SD) of the PEF value is shown in percent of the predicted PEF, scaled on age, height, weight, gender, smoker (yes, no, former), and asthma (yes, no). Means (±SD) of the 4-min post-stress recovery phase shown in percentage compared to means of the 1-min stressor phase (100% reference, indicated as *red line*)

No significant differences between the groups regarding all determined physiological parameters and peripheral signals could be detected at the beginning of the study. Exposure to the waterfall had a significant positive effect on lung function as measured via peak expiratory flow (PEF) on day 6 (WF+ to CO *p* = 0.023, *R* ^2^ = 0.346). No significant effect could be detected in the WF− group (WF− to CO *p* = 0.136, *R* ^2^ = 0.047) or between the two intervention groups after 1 week of intervention (WF+ to WF− *p* = 0.359, *R* ^2^ = 0.079) (Table 2, Fig. 7).

**Table 2:** Means of evaluated physiological data (±SD)

		PEF (L/sec)	HR (%)	SC (%)	RSP (%)
WF+	Day 0	8.7 ± 2.0	98.7 ± 5.5	88.4 ± 12.8	91.2 ± 17.3
	Day 6	9.0 ± 1.9	94.7 ± 9.2	81.5 ± 13.7	83.7 ± 16.2
WF−	Day 0	8.5 ± 1.5	97.7 ± 6.0	86.8 ± 12.4	91.1 ± 12.3
	Day 6	8.9 ± 1.7	92.0 ± 6.9	87.3 ± 14.3	82.7 ± 13.0
CO	Day 0	8.6 ± 2.1	100.6 ± 7.0	90.8 ± 8.2	96.1 ± 17.3
	Day 6	8.6 ± 1.8	101.4 ± 7.3	90.4 ± 13.6	97.4 ± 19.6

Physiological data of the intervention groups (WF+ and WF−) and the control group on day 0 and day 6. Raw data of the peak expiratory flow (PEF) shown in means (±SD). Means (±SD) of the 4-min post-stress recovery phase of heart rate (HR), skin conductance (SC), and respiration rate (RSP) are shown in percentage (in relation to the 100% reference, means of the 1-min stressor phase)

All peripheral signals of the physiological stress test decreased in the W+ and W− group after 1 week. The results show the arithmetic mean in percentage of the 4-min post-stress recovery phase, compared to a 100% reference line, which reflects the arithmetic mean of the 1-min stressor phase in percentage. Results below 100% indicate the ability to reconstitute a parasympathetic physiological state, to calm down, and to relax after a stressor within the 4-min post-stress recovery phase. Compared to the control group, the respiration rate was decreased significantly in both intervention groups (WF+ to CO *p* = 0.016, *R* ^2^ = 0.164; WF− to CO *p* = 0.004, *R* ^2^ = 0.169). Similarly, skin conductance indicating sympathetic activity and sweat production was reduced after 1 week of waterfall exposition (WF+ to CO *p* = 0.017, *R* ^2^ = 0.151), with a trend to a waterfall-specific additional effect (WF+ to WF− *p* = 0.056, *R* ^2^ = 0.187). The heart rate was significantly lowered in both intervention groups (WF+ to CO *p* = 0.011, *R* ^2^ = 0.421; WF− to CO *p* = 0.000, *R* ^2^ = 0.492) (Table 2, Fig. 7).

## Discussion

The present controlled and randomized clinical study addressed the question whether the specific environment of a waterfall provides additional beneficial effects for prophylactic and therapeutic stress management when combined with high-altitude climate therapy and physical activity (mountain hiking in the protected area National Park Hohe Tauern in Austria). As stress represents a complex phenomenon including psychological, physiological, and immunological effects, we investigated selected parameters of all of these three aspects. For this purpose, two study groups with moderate to high stress levels spent an active 1-week sojourn in the National Park Hohe Tauern with identical parameters concerning daily mountain hiking, accommodation and food supply, except for 1 h/day. One group spent this time at the waterfall, the other at the same sea level but free from the waterfall-specific environment. A third study arm was recruited as a control group, keeping their normal daily life without any intervention.

The results of our study are in line with publications indicating positive health effects of green exercise and high-altitude climate therapy, which have been already proven to contribute to the improvement of respiratory and/or allergic diseases [3–5, 16]. Our data suggest to expand the applicability of this simple and cost-effective health provision for treatment of stress-related symptoms. Interestingly, several of the investigated parameters were significantly changed with a 1-h/day exposure to the environment of a waterfall. Most strikingly, beyond psychological and physiological stress-related symptoms, the specific humoral sIgA immune response after oral vaccination was significantly improved by the additional waterfall exposure. Secretory IgA is present in all mucosal surfaces; it is of crucial importance for the first line of defense of the immune system at mucosal barriers [33, 34].

Currently, we know that the immense power of the falling water cracks small water droplets in a specific way, resulting in small nano particles carrying a negative charge, whereas the created bigger fragments are charged positively. The specific environment around waterfalls is characterized by these mainly negatively charged “Lenard’s ions” hovering in the air, while the positive-charged fragments sink to the ground [10, 60–62]. The airborne nano-aeorosol is assumed to trigger a variety of biological effects, e.g., mild activation of the immune system, inducing a balance between TH1/TH2 immunity, stabilizing the autonomous nervous system, and improving blood flow [16, 63–65]. In a mouse model, water-generated negative ions have been shown to enhance cytotoxic activity of natural killer cells [66]. It is also conceivable that the high concentration of negative air ions near waterfalls could influence the human microbiota. Furthermore, the waterfall environment itself provides a specific microbiologic atmosphere, which may directly effect the microbiota of skin and mucosal surfaces [14]. This waterfall-altered microbiome could act as an immunological adjuvant and thus be responsible for the observed effects on mucosal immunity.

Negative air ions have also been shown to influence psychologic well-being by an increased serotonin level [18] and to enhance positive emotional processing in seasonal affective disorder [67] and have also been associated with lower depression scores [68]. An “added value” concerning the mood-raising effect may simply originate from the extraordinary beauty of the waterfall itself [69].

## Conclusions

Numerous curing and healing effects are ascribed to waterfalls in ancient traditions and folk wisdom in many regions of the world. The present randomized, controlled clinical study provides evidence for an added value of a daily 1-h stay for 1 week in a waterfall environment in combination with green exercise and high-altitude climate therapy. Furthermore, the data point to an influence of the waterfall ionosols on complex “psychoneuroimmunological” regulatory circuits.

The results of this study also provides a rational basis for further research with an aim to develop schedules for new and effective prophylactic therapies for high-risk patients suffering from psychological and physiological stress symptoms.
